# A Combination of a Genome-Wide Association Study and a Transcriptome Analysis Reveals circRNAs as New Regulators Involved in the Response to Salt Stress in Maize

**DOI:** 10.3390/ijms23179755

**Published:** 2022-08-28

**Authors:** Peng Liu, Yuxiao Zhu, Hao Liu, Zhenjuan Liang, Minyan Zhang, Chaoying Zou, Guangsheng Yuan, Shibin Gao, Guangtang Pan, Yaou Shen, Langlang Ma

**Affiliations:** State Key Laboratory of Crop Gene Exploration and Utilization in Southwest China, Maize Research Institute, Sichuan Agricultural University, Chengdu 611130, China

**Keywords:** maize, salt stress, GWAS, circRNA, hub gene

## Abstract

Salinization seriously threatens the normal growth of maize, especially at the seedling stage. Recent studies have demonstrated that circular RNAs (circRNAs) play vital roles in the regulation of plant stress resistance. Here, we performed a genome-wide association study (GWAS) on the survival rate of 300 maize accessions under a salt stress treatment. A total of 5 trait-associated SNPs and 86 candidate genes were obtained by the GWAS. We performed RNA sequencing for 28 transcriptome libraries derived from 2 maize lines with contrasting salt tolerance under normal and salt treatment conditions. A total of 1217 highly expressed circRNAs were identified, of which 371 were responsive to a salt treatment. Using PCR and Sanger sequencing, we verified the reliability of these differentially expressed circRNAs. An integration of the GWAS and RNA-Seq analyses uncovered two differentially expressed hub genes (*Zm00001eb013650* and *Zm00001eb198930*), which were regulated by four circRNAs. Based on these results, we constructed a regulation model of circRNA/miRNA/mRNA that mediated salt stress tolerance in maize. By conducting hub gene-based association analyses, we detected a favorable haplotype in *Zm00001eb198930*, which was responsible for high salt tolerance. These results help to clarify the regulatory relationship between circRNAs and their target genes as well as to develop salt-tolerant lines for maize breeding.

## 1. Introduction

As one of the main abiotic stresses, excess salinity inhibits the growth, development, and yield of plants; it even threatens their survival. Salinity leads to the accumulation of Na^+^ and Cl^−^ ions and exerts a detrimental effect on plants, limiting the intake of K^+^ and Ca^2+^; this causes an ion imbalance, increasing the osmotic pressure and reducing the capacity for water absorption. To survive against salt stress, plants have developed several physiological response mechanisms, including increasing transmembrane electrochemical gradients to excrete Na^+^ from cells, synthesizing and accumulating small molecule organic compounds to balance intracellular osmotic pressure, and activating antioxidant enzymes and antioxidants to resist oxidative damage [1]. In these molecular mechanisms, the normal cell function and biological process are regulated by a number of genes or pathways such as signal transduction pathways, transcription factors, and other regulators containing long non-coding RNA (lncRNA), microRNA (miRNA), and circular RNA (circRNA) [2]. As a glycophyte, maize (*Zea mays* L.) is widely cultivated all over the world; it is sensitive to salt stress, especially at the seedling stage [3]. As such, dissecting the genetical basis and causal genes of salt tolerance in maize seedlings is helpful for cultivating salt-tolerant varieties.

Currently, a genome-wide association study (GWAS) is widely used to detect the candidate genes controlling agronomic traits. It is based on the linkage disequilibrium (LD) of molecular markers. Using a GWAS, 544 initial candidate genes affecting Ca^2+^ concentrations were detected in maize seedlings under salt stress [4]. Luo et al. identified 57 loci significantly associated with maize salt tolerance using a GWAS. According to the LD regions of these genetic loci, 49 candidate genes were finally obtained; these were involved in stress responses, ABA signaling, stomata division, DNA binding/transcription regulation, and auxin signaling [5]. Two genes, *SAG4* and *SAG6*, were further verified to regulate salt tolerance by transgenic approaches. In addition, the genes *ZmCLCg* and *ZmPMP3* were identified by a GWAS and were validated to control maize salt tolerance [6,7]. A GWAS is an efficient tool to identify the candidate genes underlying target traits, but it is not enough to accurately refine the causal genes. Conventionally, to identify the causal genes from the salt tolerance-associated candidate genes identified by a GWAS, QTL fine mapping based upon large recombinant populations and high-density markers should be carried out [8]. Owing to a high efficiency in detecting target genes, a GWAS and a transcriptome analysis have been jointly employed to identify trait-marker associations. For example, Ma et al. (2021) revealed that two hub genes, *GRMZM2G075104* and *GRMZM2G333183*, were involved in the Na^+^ and K^+^ contents in maize seedlings under salt stress conditions via a GWAS and a co-expression analysis [3]. Using a similar strategy, a seed germination ability-associated gene in maize, *MADS26*, was identified and then functionally verified by an overexpression [9].

circRNAs are a special type of endogenous RNA with a closed-loop structure; they are generated by back-splicing events in the coding and non-coding regions of the genome [10]. As the cost of high-throughput sequencing has decreased, a growing number of circRNAs have been detected in humans, animals, plants, and other species [11,12,13]. Most circRNAs contain less than seven exons and exonic circRNAs are typically shorter than 600 bp in length [13,14]. The expression abundance of circRNAs displays a spatiotemporal specificity [15]. circRNAs regulate the expression of functional genes by acting as an miRNA sponge or by influencing the splicing of their linear counterparts [13,16]. Previous studies have shown that circRNAs exert important roles in signal transcription, metabolism adaptation, and ion homeostasis under salt and dehydration stresses in cucumbers [17], rice [18], wheat [19], sugar beet [20], and poplars [21]. However, there are only a few reports on circRNAs involved in the response to abiotic stresses in maize [22].

In this study, we detected the circRNAs that responded to salt stress based on the whole transcriptome sequencing data from two maize inbred lines with contrasting salt tolerance. Subsequently, we performed a GWAS to identify the candidate genes associated with the survival rate (SR) under salt stress. When combining the results of the circRNAs and GWAS, we found that two hub genes (*Zm00001eb013650* and *Zm00001eb198930*) were regulated by the circRNAs. A candidate gene association analysis revealed a salt-tolerant haplotype for the hub gene *Zm00001eb198930*. Finally, we constructed regulation models of *Zm00001eb013650* and *Zm00001eb198930*. These results are useful to understand the genetic control of maize salt tolerance and to accelerate the application of molecular marker-assisted selection in maize breeding.

## 2. Results

### 2.1. Identification, Characterization, and Validation of circRNAs in Maize Seedlings

**Figure 1:**
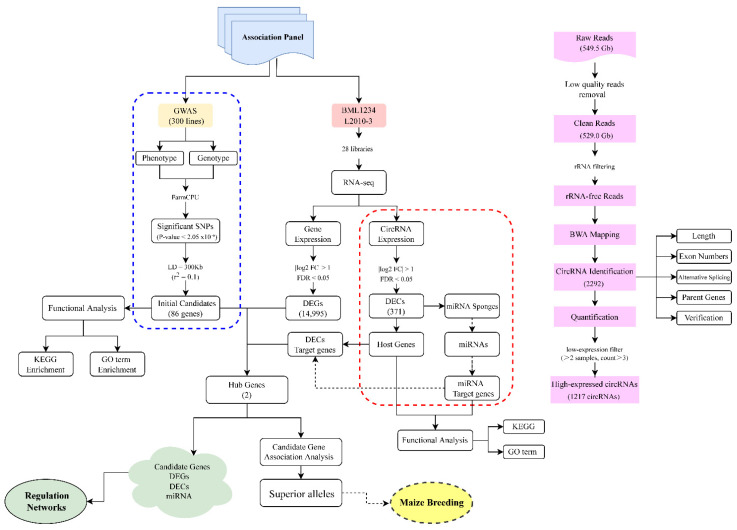
The workflow of the bioinformatic analysis used in this study. Pink rectangular boxes on the right side show the main steps of circRNA identification. The blue dotted box contains the main steps of the genome-wide association study (GWAS). The red dotted box contains the main steps of the circRNA study. LD represents the linkage disequilibrium. KEGG represents the Kyoto Encyclopedia of Genes and Genomes. GO represents the gene ontology. DEGs represents differentially expressed genes. DECs represents differentially expressed circRNAs. The green cloud shape represents the members used to construct regulation network. The yellow ellipse represents the goal of this study.

**Figure 2:**
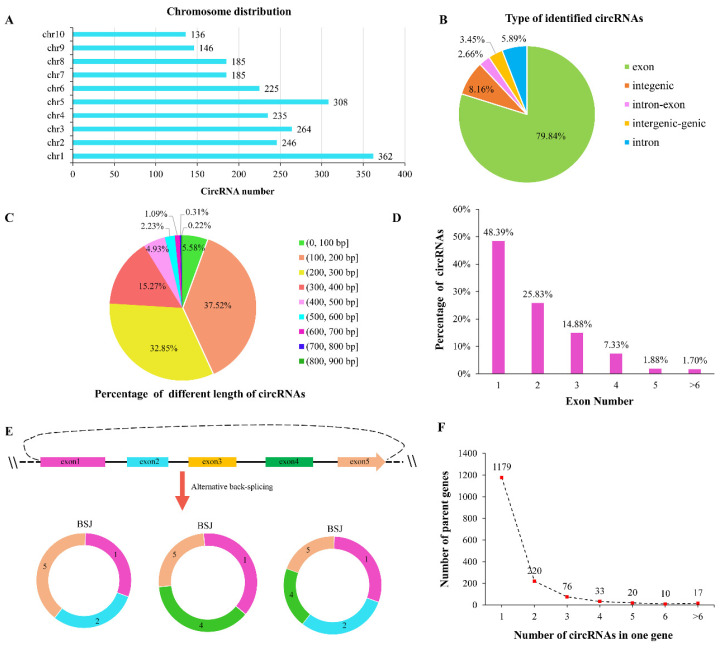
Characteristics of identified circRNAs. (**A**) The distribution of circRNAs on different chromosomes. (**B**) The distribution of different circRNA types. (**C**) Percentage of circRNAs of different lengths. (**D**) Percentage of circRNAs containing different numbers of internal exons. X-axis represents the internal exon numbers of circRNAs. Y-axis represents the percentage of circRNAs. (**E**) Multiple types of circRNAs generated via alternative back-splicing. (**F**) The number of genes generating different numbers of circRNAs. X-axis represents the number of circRNAs generated from one gene. Y-axis represents the number of genes.

In the present study, two maize lines with contrasting salt tolerance were selected from an association panel to perform the transcriptome analysis. In total, 529.5 Gb of raw sequencing data were obtained from 28 RNA-Seq libraries, which consisted of 4 treatment phases (0 h, 6 h, 18 h, and 36 h) and 2 biological repetitions. The detailed descriptions of each sample are shown in Table S1. Using a rigorous pipeline (Figure 1), a total of 2292 circRNAs were detected in maize seedlings under normal and salt treatment conditions. Detailed information on the identified circRNAs are shown in Table S2. These circRNAs were distributed on 10 chromosomes. Chromosome 1 (Chr1) included the greatest number of circRNAs, followed by Chr5, Chr3, and Chr2 (Figure 2A). According to the maize reference annotations (https://maizegdb.org/genome/assembly/Zm-B73-REFERENCE-NAM-5.0 (accessed on 13 November 2021)), the circRNAs were classified into five groups. The majority (1830, 79.84%) of these circRNAs were generated from the exon regions and named as exonic circRNAs, followed by intergenic and intronic circRNAs (187 (8.16%) and 135 (5.89%), respectively). A total of 79 (3.45%) and 61 (2.66%) circRNAs were identified as intergenic-genic and intron-exon circRNAs, respectively (Figure 2B).

We then analyzed the internal compositions of these full-length circRNAs. Most circRNAs (98.39%) were shorter than 600 bp in length (Figure 2C). Approximately 50% of the circRNAs were composed of only one exon and more than 89% of the circRNAs contained less than four internal exons. However, only ten (1.7%) circRNAs contained more than six exons (Figure 2D). Multiple circRNAs were derived from the same back-splice site in a single gene by alternative back-splicing (Figure 2E). In this study, 2035 unique back-splice junctions (BSJ) were detected in all the circRNAs. In total, 164 BSJs had more than 1 alternative back-splicing circularization event. More than 91% (2106) of the circRNA transcripts were produced by 1555 parent genes. A total of 376 specific parent genes produced more than 1 circRNA (Figure 2F). For example, 22 circRNAs originated from the gene *Zm00001eb269100*, which is involved in DNA binding, ribonucleoside binding, and metal ion binding.

**Figure 3:**
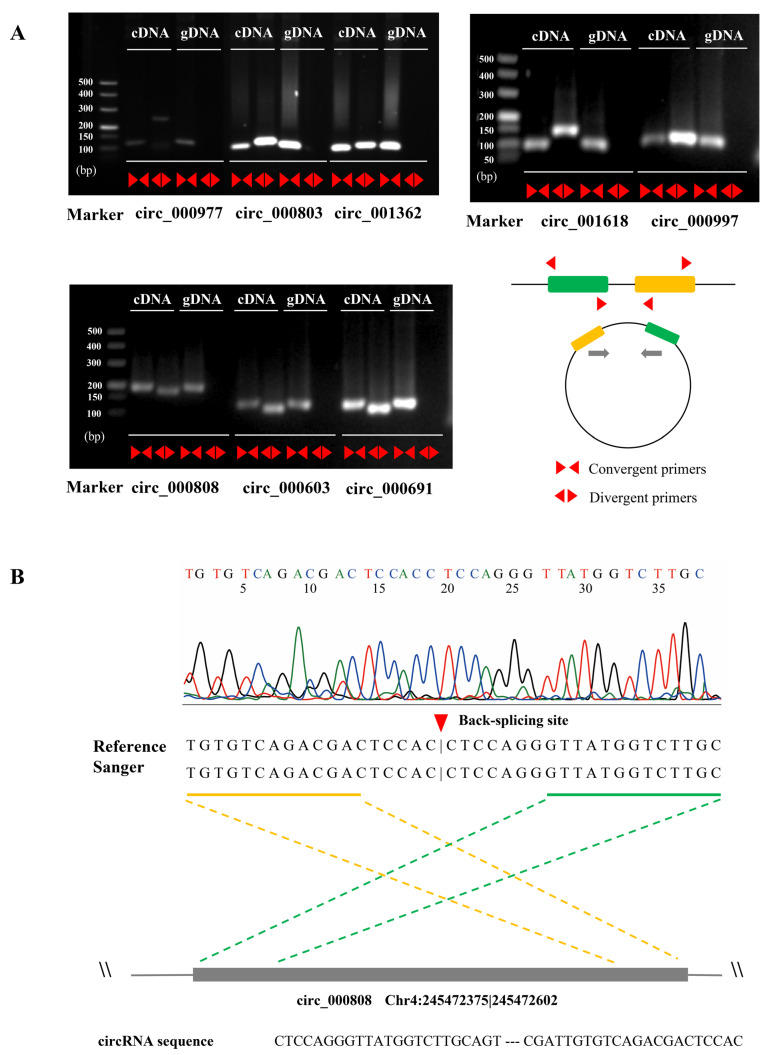
Validation of circRNAs. (**A**) PCR amplification for eight circRNAs. (**B**) Sanger sequencing for circ_000808. The head-to-tail splicing of circ_000808 was confirmed by Sanger sequencing. The red triangle arrow represents the back-splicing site.

We evaluated the expression levels of the circRNAs in different samples and identified 1217 putative circRNAs with high expressions. Among them, approximately 79% had median TPM (transcripts per million) values of <10 among the 28 samples. Only 36.07% (439) circRNAs were simultaneously expressed in all the samples. These results proved the specific expression patterns of the circRNAs and were consistent with previous studies in maize [23]. Subsequently, we randomly selected ten circRNAs for an experimental validation. As a result, the facticity of eight circRNAs were validated by PCR amplification (Figure 3A,B). In addition, the expression levels of four randomly selected circRNAs from the RNA-Seq were well-matched with the results of the real-time quantitative reverse transcription PCR (qRT-PCR) (R^2^ > 0.6) (Figure S1). This evidence indicated that the identified circRNAs were accurate.

### 2.2. Differentially Expressed circRNAs and Genes Induced by Salt Stress

**Figure 4:**
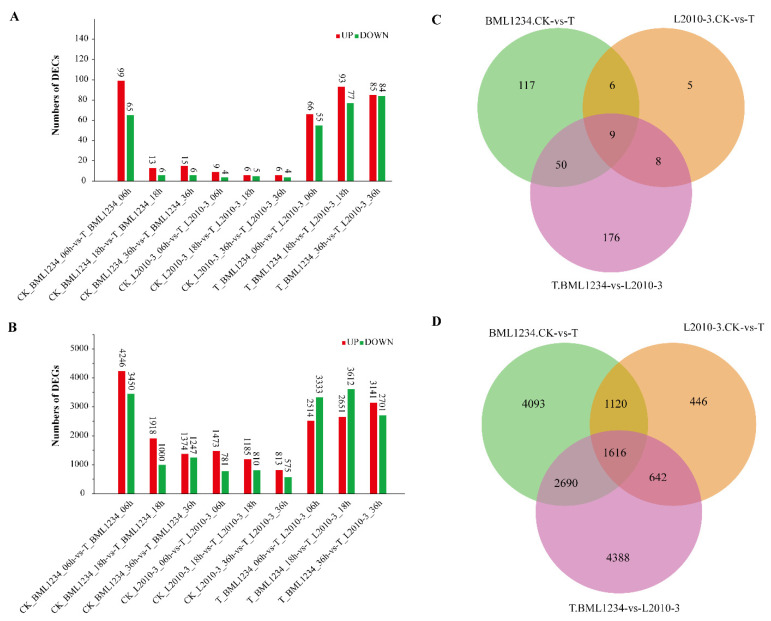
Statistics of differentially expressed circRNAs and genes. (**A**,**B**) represent the number of up- and downregulated circRNAs and genes, respectively, in different pairwise comparison groups. (**C**,**D**) represent the number of differentially expressed circRNAs (DECs) and genes (DEGs), respectively, in different comparison groups.

To reveal the effect of salt stress on maize circRNAs and genes, we compared the expressions of nine pairwise samples, including CK_BML1234_06h vs. T_BML1234_06h, CK_BML1234_18h vs. T_BML1234_18h, CK_BML1234_36h vs. T_BML1234_36h, CK_L2010-3_06h vs. T_L2010-3_06h, CK_L2010-3_18h vs. T_L2010-3_18h, CK_L2010-3_36h vs. T_L2010-3_36h, T_BML1234_06h vs. T_L2010-3_06h, T_BML1234_18h vs. T_L2010-3_18h, and T_BML1234_36h vs. T_L2010-3_36h, respectively. Under |log2 FC| > 1 and FDR < 0.05 conditions, 371 differentially expressed circRNAs (DECs) and 14,995 differentially expressed genes (DEGs) were detected in all comparison groups (Figure 4A,B and Tables S3 and S4). Among the DECs, 243 were found in the comparison between different materials, implying that these two lines were different. Under salt treatment conditions, we found 182 DECs in the salt-sensitive line BML1234, including 164 (99 up- and 65 downregulated) DECs at 6 h, 19 (13 up- and 6 downregulated) DECs at 18 h, and 21 (15 up- and 6 downregulated) DECs at 36 h (Figure 4A). Only 29 DECs were detected in the salt-tolerant line L2010-3, including 13 (9 up- and 4 downregulated), 10 (5 up- and 5 downregulated), and 10 (6 up- and 4 downregulated) DECs at 6, 18, and 36 h, respectively (Figure 4A). Additionally, a greater number of DEGs were detected in the salt-sensitive line than in the salt-tolerant line, especially at 6 h hour after salt stress, which had a consistent trend in comparison with that of the DECs (Figure 4C,D). The heatmap based on the change fold of each DEC is shown in Figure S2. The expression patterns of nine common DECs are shown in Figure S3. These results suggested that the expressions of circRNAs and protein-coding genes were more easily affected by environmental stresses at an earlier stage.

To further explore the biological function of these DECs, we predicted their target genes. Finally, a total of 1860 genes were identified as circRNA target genes, including 150 circRNA host genes and 1720 miRNA target genes. The Gene Ontology (GO) enrichment analysis indicated that the 150 host genes were mainly involved in various biological processes such as the glutamine metabolic process (GO:0006541), response to oxidative stress (GO:0006979), and response to a xenobiotic stimulus (GO:0009410). The Kyoto Encyclopedia of Genes and Genomes (KEGG) pathway enrichment analysis revealed that two pathways, “alanine, aspartate and glutamate metabolism” and “biosynthesis of amino acids”, were significantly enriched, suggesting that several circRNAs were involved in the response to salt stress by participating in the regulation of amino acid metabolism. In a previous study, the amino acid metabolic pathway was reported to play a vital role in the process of plant adaptation to salt stress; our findings supported this observation [24]. In addition, 1720 target genes were regulated by 25 DECs via 30 miRNAs. Most of these miRNA families have previously been reported to respond to salt stress [25,26], including zma-miR160, zma-miR164, and zma-miR399. The GO categories of these genes showed that most GO terms were enriched in the molecular function category as “monooxygenase activity” (GO:0004497), “sarcosine oxidase activity” (GO:0008115), and “protein kinase activity” (GO:0004672). The KEGG enrichment analysis showed that the significantly enriched pathways of these targets were “peroxisome”, “synthesis and degradation of ketone bodies”, “metabolic pathways”, and “flavonoid biosynthesis”. In addition, 1146 of the 1860 target genes were detected as DEGs, of which 6 belong to the host genes and miRNA target genes.

### 2.3. GWAS of Maize Salt Tolerance

**Figure 5:**
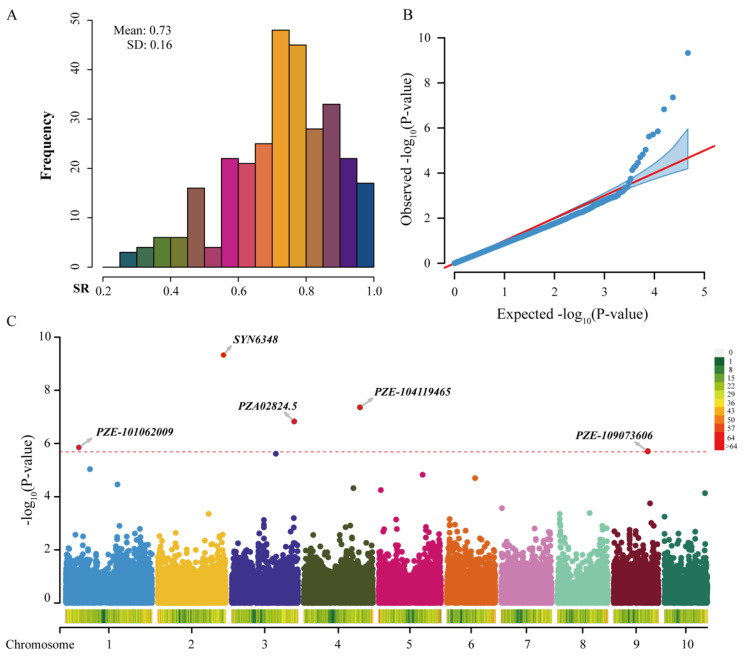
Distribution of SR and significant associations with SR under salt stress. (**A**) The phenotype distribution of SR under salt stress. (**B**) QQ plot of the GWAS by FarmCPU. (**C**) Manhattan plot of the GWAS. The dashed line represents the significance threshold (*p*-value = 2.05 × 10^−6^).

In this study, we investigated the survival rate values of 300 lines to estimate the salt tolerance of different genotypes. Among these inbred lines, the SR ranged from 27.78% to 100%, with a mean value of 73.18%. The standard deviation (SD) and coefficient of variation (CV) were 0.16 and 21.80%, respectively, suggesting that the SR presented abundant variations among the different lines. In addition, the phenotypes of the SR followed a normal distribution, suggesting that the SR was a quantitative trait (Figure 5A).

To determine the optimal model for the association analysis, a general linear model (GLM), a mixed linear model (MLM), and a fixed and random model circulating probability unification (FarmCPU) were compared though quantile–quantile (QQ) plots (Figure S4). For the FarmCPU model, the QQ plot led to a sharp deviation from the expected *p*-values, especially in the tail area. This verified that the FarmCPU effectively governed false-positives and -negatives and was suitable for the GWAS of the SR. Using the mean values of three biological repetitions as the phenotypic data, five trait-associated SNPs with a *p*-value < 2.05 × 10^−6^ (0.05/24360) were detected by the FarmCPU model in the rMVP package (Table 1).

The phenotypic variation explained (PVE) by a single SNP ranged from 0.75% to 5.79%. Among these SNPs, SYN6348 (*p*-value = 4.71 × 10^−10^) was the lead SNP; it was located in chromosome 2 and explained the 4.41% phenotypic variation. This SNP was in the first intron of the gene *Zm00001eb115230*, which encodes a ribosome-associated membrane protein (RAMP4). A member of the RAMP4 family in *Brassica juncea*, *BjJ10-2*, was overexpressed in *E. coli*, resulting in an increased salt tolerance in the recombinant *E. coli* strain [27]. A significant SNP, PZE-104119465, was located in the ninth intron of the gene *Zm00001eb198950*; this encodes an exocyst subunit Exo70 family protein. Interestingly, several members of this gene family have been reported to be upregulated under salt stress [28,29]. PZA02824.5 was located in the fourth exon of gene *Zm00001eb159890*; this was annotated as an early flowering 3 (ELF3) protein. *ELF3* has been shown to increase salt tolerance by suppressing salt stress response pathways in Arabidopsis [30] and soybeans [31]. PZE-101062009 resided in the first exon of the gene *Zm00001eb013540*. This gene encodes a tetratricopeptide repeat (TPR)-like superfamily protein, which is involved in the regulation of different cellular functions [32,33]. In tomatoes, TPR family genes were previously reported to respond to various biotic and abiotic stresses [34].

A total of 86 genes were located in the LD = 300 kb regions around these significant SNPs and they were selected as the initial candidate genes (Table S5). Around the LD regions of the lead SNP, SYN6348, 22 candidate genes were found. Among these, the gene *Zm00001eb115140*, which encodes a patatin-like phospholipase protein, has previously been reported to be involved in osmotic and salt tolerance in Arabidopsis [35]. The genes *Zm00001eb115320* and *Zm00001eb115330*—which encode a late embryogenesis abundant (LEA) protein and a NAD-dependent epimerase/dehydratase family protein, respectively—have been reported to participate in response to abiotic stresses [36,37,38]. In addition, 23 candidate genes were detected for the SNP PZE-101062009 (*p*-value = 1.41 × 10^−6^), including *Zm00001eb013450* (protein phosphatase 2C), *Zm00001eb013460* (mitochondrial carrier protein), *Zm00001eb013480* (protein kinase), *Zm00001eb013490* (RanBP1 domain), *Zm00001eb013520* (thiamine pyrophosphate enzyme), *Zm00001eb013550* (6-phosphofructo-2-kinase), *Zm00001eb013560* (late embryogenesis abundant protein), *Zm00001eb013590* (Myb/SANT-like DNA binding domain), *Zm00001eb013610* (protein tyrosine kinase), and *Zm00001eb013650* (protein tyrosine kinase), which have been reported to correlate with the response to salt stress [39,40,41,42,43,44]. To further recognize the function of these initial candidate genes, we performed a GO term enrichment analysis. In the GO categories of biological processes, 15 GO terms were significant enrichments with a *p*-value < 0.01. Five terms—“response to stimulus”, “tricarboxylic acid transport”, “fumarate transport”, “citrate transport”, and “antibiotic transport”—have been reported to respond to salt stress (Figure S5, Table S6).

We identified three salt tolerance-related circRNAs, *circ_000260*, *circ_001362*, and *circ_001730*, which originated from *Zm00001eb013550* (6-phosphofructo-2-kinase), *Zm00001eb198930* (non-coding gene), and *Zm00001eb198990* (UBX domain protein), respectively. Intriguingly, the 6-phosphofructo-2-kinase protein and UBX domain protein in rice were significantly upregulated under salt stress [45,46].

### 2.4. Combination of the GWAS and RNA-Seq to Identify circRNA-Mediated Hub Genes in Response to Salt Stress

To further identify the circRNA-mediated hub genes affecting salt tolerance, we focused on the common genes simultaneously detected by the GWAS and RNA-Seq analysis. Of the 86 candidate genes detected by the GWAS, 33 (6 non-coding and 27 protein-coding) genes were differentially expressed. Among the 27 protein-coding candidates, 3 genes (*Zm00001eb115140*, *Zm00001eb115150*, and *Zm00001eb115160*), 2 genes (*Zm00001eb013450* and *Zm00001eb115340*), and 1 gene (*Zm00001eb198960*) correlated with the lipid metabolic process, dephosphorylation, and rhythmic process, respectively. As a dephosphorylation-related gene, *Zm00001eb013450* encodes a phosphatase 2C protein, which has been widely reported to respond to multiple stresses (including sugar, cold, drought, and salt) in plants [47,48,49,50].

Among the 33 common genes, *Zm00001eb013650* (protein-coding) and *Zm00001eb198930* (non-coding) were identified as the target genes of 4 circRNAs. *Zm00001eb013650* was targeted by three miRNA sponges (*circ_000078*, *circ_001169*, and *circ_001523*) that were mediated by two miRNAs (*zma-miR399g-5p* and *zma-miR160f-3p*). *Zm00001eb013650* was annotated as a tyrosine kinase, which plays an important role in various abiotic stress responses [51]. Under salt stress conditions, the expression levels of *Zm00001eb013650* and two regulators, *circ_000078* and *circ_001169*, were all higher in L2010-3 than in BML1234. In BML1234, the expression level of the regulator *circ_001169* was significantly increased at 6 h of salt treatment relative to that under normal conditions. *Zm00001eb198930* was identified as the host gene of *circ_001362*. The expression abundances of *Zm00001eb198930* and *circ_001362* were both significantly higher in BML1234 at 6 h of salt treatment than those under normal conditions. The expression patterns of these four circRNAs and two hub genes are shown in Figure S6.

### 2.5. Intragenic Variations in Hub Genes Affecting the SR under Salt Stress

**Figure 6:**
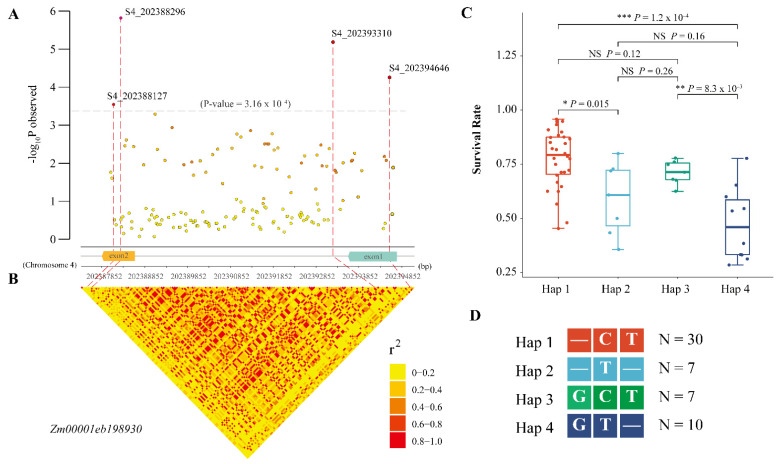
Candidate gene association analysis of *Zm00001eb198930*. (**A**) Significant SNPs/indels associated with SR. X-axis represents the genomic position on chromosome 4. The structure of *Zm00001eb198930* is displayed in the middle. (**B**) Pairwise linkage disequilibrium between the markers. (**C**) Comparison of SR between different haplotypes. * Significant at *p* < 0.05. ** Significant at *p* < 0.01. *** Significant at *p* < 0.001. NS = not significant. (**D**) Details of four haplotypes. “—” represents a deletion. N represents the inbred line number of each haplotype.

To identify the key intragenic variants affecting the SR, we randomly selected 69 inbred lines from the maize panel and performed candidate gene association analyses for the 2 hub genes *Zm00001eb013650* and *Zm00001eb198930*. Finally, we detected 70 (55 SNPs and 15 indels) and 158 (114 SNPs and 44 indels) genetic variations in *Zm00001eb013650* and *Zm00001eb198930*, respectively. Using the GLM model, we found that only one synonymous variant in *Zm00001eb013650* was significantly associated with the SR with a *p*-value < 7.14 × 10^−4^. Four significant variants of *Zm00001eb198930* with a *p*-value < 3.16 × 10^−4^ were associated with the SR, including three exon variants and one intron variant (Figure 6A,B). Based on the 3 exon variants of *Zm00001eb198930*, the 69 lines were classified into 4 major haplotypes (Hap 1: —CT; Hap 2: —T—; Hap 3: GCT; and Hap 4: GC—). Among these haplotypes, Hap 1 showed a significantly higher SR than each of the other haplotypes. Thus, the Hap 1 of *Zm00001eb198930* was confirmed as the favorable haplotype for salt tolerance (Figure 6C,D).

## 3. Discussion

### 3.1. circRNAs Play Important Roles in Response to Salt Stress in Maize

As a type of covalently closed single-stranded RNA, circRNAs are widespread in eukaryotes. During the past decades, a large number of circRNAs have been discovered in various plants with the development of sequencing and bioinformatics technology. In total, 43,883 and 52,393 circRNAs have been identified in rice and Arabidopsis, respectively, in PlantCircBase (http://ibi.zju.edu.cn/plantcircbase/index.php (accessed on 13 July 2022)). In maize, 10,381 and 38,785 circRNAs have been separately identified in the PlantCircBase and CropCircDB (http://deepbiology.cn/crop/index.php (accessed on 17 August 2022)) databases, respectively; these were mainly grouped into exonic circRNAs. Most circRNAs have been reported to regulate plant growth, development, and stress responses. For example, under a low-temperature treatment, 1759 circRNAs were induced in tomato leaves [52]. In maize, a total of 24 and 22 DECs obtained from the leaves and roots, respectively, were responsive to deficient nitrogen stress [53]. However, knowledge of the roles of circRNAs in response to salt stress are still limited in maize. In the present study, we constructed full-length sequences of 2292 potential circRNAs for maize seedlings under a salt treatment using 2 contrasting salt-sensitive lines. By PCR and Sanger sequencing, eight of ten randomly selected circRNAs were verified. Most of the identified circRNAs were generated from the exon regions of the genome, consistent with the results from previous studies. The functions of exonic circRNAs usually correlate with their host genes. In addition, circRNAs can act as miRNA sponges, regulating their corresponding miRNA targets. Herein, we analyzed 150 host genes and 1720 miRNA target genes for 371 salt stress response-related DECs. According to the functional annotations, these target genes were involved in multiple biological processes and pathways such as the cellular amino acid metabolic process, response to a xenobiotic stimulus, regulation of seedling development, and signaling. These results provide a basis to further understand the biological function of maize circRNAs under salt stress.

### 3.2. An Effective Method to Identify Causal Genes by Combining a GWAS and Transcriptome Analysis

A GWAS has been widely used to detect potential salt tolerance-related genes in maize. Using 557,894 SNPs of 384 maize lines, 13 candidate genes were associated with salinity tolerance [7]. However, due to false-positives and large-scale associations, a GWAS cannot refine the causal genes controlling the target traits, which requires researchers to combine other approaches such as a transcriptome analysis and linkage mapping. The combined use of a GWAS and a transcriptome analysis has been proven to be a promising strategy for determining the key genes of target traits. Using this method, 39 and 13 salt-responding genes have been identified in barley [54] and cotton [55], respectively. In the present study, we detected 86 candidate genes and 3 circRNAs around the 5 SR-associated SNPs using a GWAS, of which 33 genes and 1 circRNA were differentially expressed in the RNA-Seq. Among these common genes, *Zm00001eb013450* encoded a phosphatase 2C protein, which is a core component of the abscisic acid signal pathway. In maize, by overexpressing a member of the protein phosphatase 2C (PP2C) gene family, *ZmPP2C55*, its tolerance to drought stress was positively enhanced [56]. In tobacco, the overexpression of *ZmPP2C2* improved its tolerance to low temperatures [57]. In the present study, *Zm00001eb115320* and *Zm00001eb198910* were identified as two members of the *ZmLEA* gene family. As a large group of polypeptides, the maize LEA family members had different expression patterns under various abiotic stresses [58]. In Arabidopsis, the overexpression of the maize LEA family member *Rab 17* resulted in an increased tolerance to high salinity in transgenic plants [59]. All these findings support the credibility of the salt response-related genes identified in this study.

### 3.3. circRNA-Mediated Regulatory Model under Salt Stress in Maize

**Figure 7:**
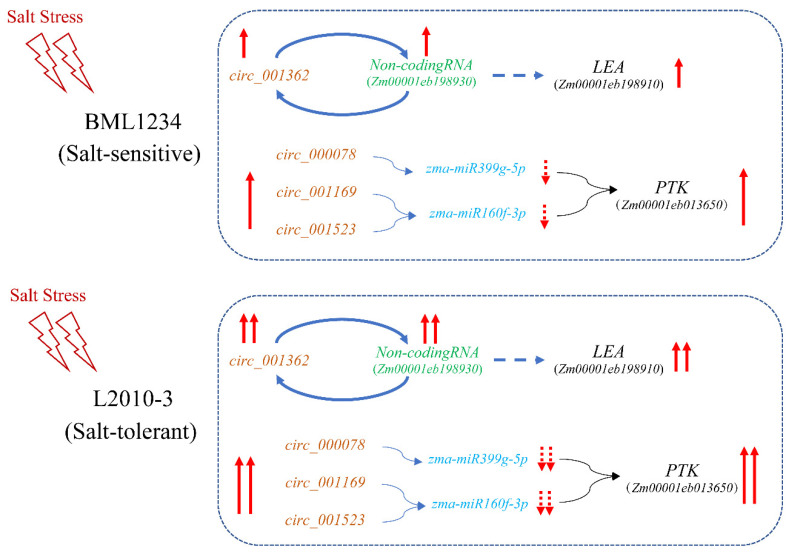
circRNA-mediated molecular regulation model.

To date, increasing evidence suggests that the interactions between circRNAs and mRNAs affect the tolerance of plants under abiotic stresses [20,60,61]. It has been proven that circRNAs can regulate the gene expression by altering the splicing of their host genes or competing with their target genes to absorb their corresponding miRNAs at post-transcriptional levels [62]. Several well-known miRNA families of miR160 and miR399 are involved in a salt stress response [63,64]. In this study, we used a comprehensive workflow to investigate the roles of circRNAs in responding to salt stress (Figure 1) and found two hub candidate genes regulated by circRNAs in different patterns. In addition, *Zm00001eb198930* cis-regulated the expression of a downstream gene, *Zm00001eb198910*. Based on these results, we constructed two models to explain the circRNA-mediated regulatory networks in the salt stress response (Figure 7). Under salt stress, the upregulated expression of *circ_001362* resulted in an increase in its host gene. As a cis-acting regulator, highly expressed *Zm00001eb198930* promoted the accumulation of the LEA protein and then improved the tolerance to salt stress. Meanwhile, highly expressed miRNA sponges (*circ_000078*, *circ_001169*, and *circ_001523*) facilitated the adsorption of *zma-miR160f-3p* and *zma-miR399g-5p*, thereby inhibiting the splicing of the target gene by miRNAs and increasing the tolerance to salt stress. However, the facticity of the identified circRNAs in our study should be further validated to support the putative regulation networks of these four circRNAs. This study has expanded our knowledge about the characteristics of maize circRNAs and the regulating diversity. This will help us to fully understand the mechanism of circRNAs in responding to salt stress.

## 4. Materials and Methods

### 4.1. Data Preparation for the Transcriptome Analysis

Two maize inbred lines, BML1234 (a salt-sensitive line) and L2010-3 (a salt-tolerant line), were selected from the association panel for the transcriptomic analysis. The transcriptome data of 28 sequencing libraries were composed of the roots under a control and salt treatment conditions at different stages (0, 6, 18, and 36 h) as described in our previous studies [65,66]. The dataset can be downloaded from NGDC under the accession number CRA003872.

Clean reads were obtained from the raw reads by the removal of the reads with a low quality as well as polyN-containing or adapter sequences via the fastp (version: 0.23.1, HaploX Biotechnology, Shenzhen, China) program [67]. Potential rRNA reads were deleted by mapping the clean reads to the plant rRNA database (RNACentral v19, accessed on 13 November 2021) using bowtie2 (version: 2.4.4, Johns Hopkins University, Baltimore, MD, USA) software [68]. The remaining rRNA-depleted reads were used for circRNA identification and the expression pattern analysis.

### 4.2. circRNA Identification and Differential Expression Analysis

Considering that low-abundance circRNAs existed in the rRNA-depleted transcriptome data, we used a comprehensive strategy to identify the circRNAs. First, we merged and de-duplicated the rRNA-depleted reads of each biological repetition library under the same conditions. We then mapped the reads to the maize reference genome (https://maizegdb.org/genome/assembly/Zm-B73-REFERENCE-NAM-5.0 (accessed on 13 November 2021)) with BWA (version: 0.7.17-r1188, Heng Li, Boston, MA, USA) software [69]. CIRI2 (version: 2.0.6, Yuan Gao, Beijing, China) [70], CIRI-full (version: 2.1.1, Yi Zheng, Beijing, China) [71], and CIRI-vis (version: 1.4, Yi Zheng, Beijing, China) [72] tools were used to detect the circular isoforms and reconstruct the full-length sequences. Finally, a non-redundant dataset containing all circRNAs was created using a custom Perl script.

The expression level of each circRNA was identified using the psirc-quant program [73] based on likelihood maximization by mapping the rRNA-depleted reads to the integrated circRNAs. The TPM was calculated to normalize the expression level of each circRNA by the following formula:

$$\mathrm{TPM}=\frac{\mathrm{reads}\;\mathrm{mapped}\;\mathrm{to}\;\mathrm{isoform}/\mathrm{isoform}\;\mathrm{length}}{\mathrm{sum}\left(\mathrm{reads}\;\mathrm{mapped}\;\mathrm{to}\;\mathrm{isoform}/\mathrm{isoform}\;\mathrm{length}\right)}\times 10^{6}.$$

circRNAs with a count number > 3 in at least three libraries were considered to be highly expressed circRNAs [74]. The DECs and DEGs were detected by the EdgeR package in R [75] under |log2 FC| > 1 and FDR < 0.05 criteria.

### 4.3. Validation of the circRNAs

To confirm the reliability of the predicted circRNAs in maize, we designed divergent and convergent primers for ten randomly selected circRNAs from highly expressed DECs. First, total DNA and RNA were extracted from two maize lines, BML1234 and L2010-3, under control and salt stress conditions. cDNA (treated by RNase R (ZhongBei LinGe Biotechnology Ltd., Beijing, China)), cDNA, and DNA were the separately used as the templates for PCR amplification with the programs as follows: 95 °C for 3 min for 1 cycle, 95 °C for 15 s, 60 °C for 15 s, and 72 °C for 60 s for 35 cycles, and 72 °C for 5 min, followed by a hold at 12 °C. All PCR products were validated by Sanger sequencing (Tsingke Biotechnology Co., Ltd., Beijing, China). 

qRT-PCR was used to assess the relative expression of the DECs by a Bio-Rad CFX Connect system. In total, four circRNAs were randomly selected to execute the experimental validation. The *ZmUBQ* (*Zm00001eb203340*) gene was used as an internal reference gene. The relative expression value was calculated by the 2^−^^△△Ct^ method [66]. All primers used in this study are listed in Table S7.

### 4.4. Target Gene Prediction of the circRNAs

The target genes of circRNAs contain their host genes and the targets of their sponge miRNAs [13]. The miRNA binding sites in circRNA sequences were predicted using the miRanda [76] and targetfinder [77] programs. Based on the expression levels, the predicted target genes correlating with their corresponding circRNAs under the R > 0.4 and *p*-value < 0.05 conditions were identified as the high-confidence target genes of the circRNAs. The GO term enrichment and KEGG pathway analysis were performed using the OmicShare platform (https://www.omicshare.com/tools (accessed on 1 June 2022)).

### 4.5. Plant Materials, Phenotypic Data Collection, and Statistical Analysis

In this study, we used an association panel for the GWAS, which comprised 300 inbred lines [78]. To investigate the SR of the maize seedlings under salt stress, 30 maize seeds of each line with a uniform size were selected and germinated on filter paper saturated with distilled water until the two-leaf stage. The seedlings were then cultured into Hoagland’s solution, with 150 mM NaCl for the salt stress under a randomized complete block design. The trail was performed by three biological repetitions for each line. The SR was calculated on the seventh day using the following formula:

$$\mathrm{SR}=\frac{\mathrm{Number}\;\mathrm{of}\;\mathrm{surviving}\;\mathrm{seedlings}}{\mathrm{Total}\;\mathrm{number}\;\mathrm{of}\;\mathrm{seedlings}}\times 100\%.$$

The statistical indicators included the mean value, SD, maximum value, and minimum value. The mean value across the three biological repetitions was used as the phenotypic value for the GWAS.

### 4.6. GWAS for the SR of Maize Seedlings under Salt Stress

The genotype of the association panel was detected by Illumina MaizeSNP50 BeadChip in our previous study [78]. After removing the SNPs with a minor allele frequency (MAF) < 0.05, a missing rate > 20%, or heterozygosity > 30%, a total of 46,483 SNPs remained, which were evenly distributed over 10 chromosomes. Considering the effects of population stratification and kinship, three models (GLM, MLM, and FarmCPU) were tested in R with the rMVP package [79,80]. Based on the results of the QQ plots, the FarmCPU model was selected as the optimal model for the GWAS. A *p*-value of 2.05 × 10^−6^ was used to determine the significant associations and was calculated by the formula *p*-value = 0.05/N, where N (24,360) represented the number of effective SNPs calculated by the simpleM program in R [81]. All genes located in the LD regions around each significant SNP were considered as initial candidate genes. The mean LD decay across all chromosomes was approximately 300 kb at an r^2^ = 0.1 [82]. The estimation of the PVE in the FarmCPU was calculated by the linear regression method [83].

### 4.7. Candidate Gene Association Study

The variants located within the gene body and upstream of each candidate gene were collected by DNA-seq from 69 inbred lines, which were randomly selected from the association panel. A custom Perl script was used to filter out the variants with a MAF < 0.05, a missing rate > 0.8, or a heterozygosity rate > 0.3. All retained variations and phenotypic data were combined to conduct an association analysis using TASSEL (version: 5.2.84, Peter J. Bradbury, New York, NY, USA) software [84] based on the GLM. A *p*-value = 0.05/N (N stands for the number of variants in each gene) was defined as the cutoff to select the significantly associated variants for the target traits. The pairwise linkage disequilibrium between the variants was calculated by the IntAssoPlot package (build in R 4.1.3, Fengyu He, Jingzhou, China) [85] in R. The haplotypes with a higher SR were defined as superior haplotypes. The Kruskal–Wallis test of the SR among the different haplotypes was performed in R.

## Figures and Tables

**Table 1:** Significantly associated SNPs detected by the FarmCPU model.

Markers	Chr	Position	Allele	Effect	SE	PVE	*p*-Value
SYN6348	2	229,479,599	A/G	−0.0396	0.0067	4.41%	4.71 × 10^−10^
PZE-104119465	4	196,370,737	G/A	0.0362	0.0072	5.79%	4.39 × 10^−8^
PZA02824.5	3	218,898,682	G/A	0.0424	0.0090	1.83%	1.48 × 10^−7^
PZE-101062009	1	45,633,273	A/C	−0.0288	0.0067	4.40%	1.41 × 10^−6^
PZE-109073606	9	118,923,900	C/A	−0.0347	0.0083	0.75%	1.95 × 10^−6^

Chr, chromosome. SE, standard error. PVE, phenotypic variation explained.

## Data Availability

All data generated or analyzed during this study are available within the article or upon request from the corresponding author.

## Supplementary Materials

The following supporting information can be downloaded at: https://www.mdpi.com/article/10.3390/ijms23179755/s1.
