# Supplementary material for: A Combination of a Genome-Wide Association Study and a Transcriptome Analysis Reveals circRNAs as New Regulators Involved in the Response to Salt Stress in Maize
Source: Int J Mol Sci. 2022 Aug 28;23(17):9755. doi: 10.3390/ijms23179755 (PMC9456493; doi:10.3390/ijms23179755)
Supplement: Supplementary file 1 [file ijms-23-09755-s001.zip › Figure S5.pdf]

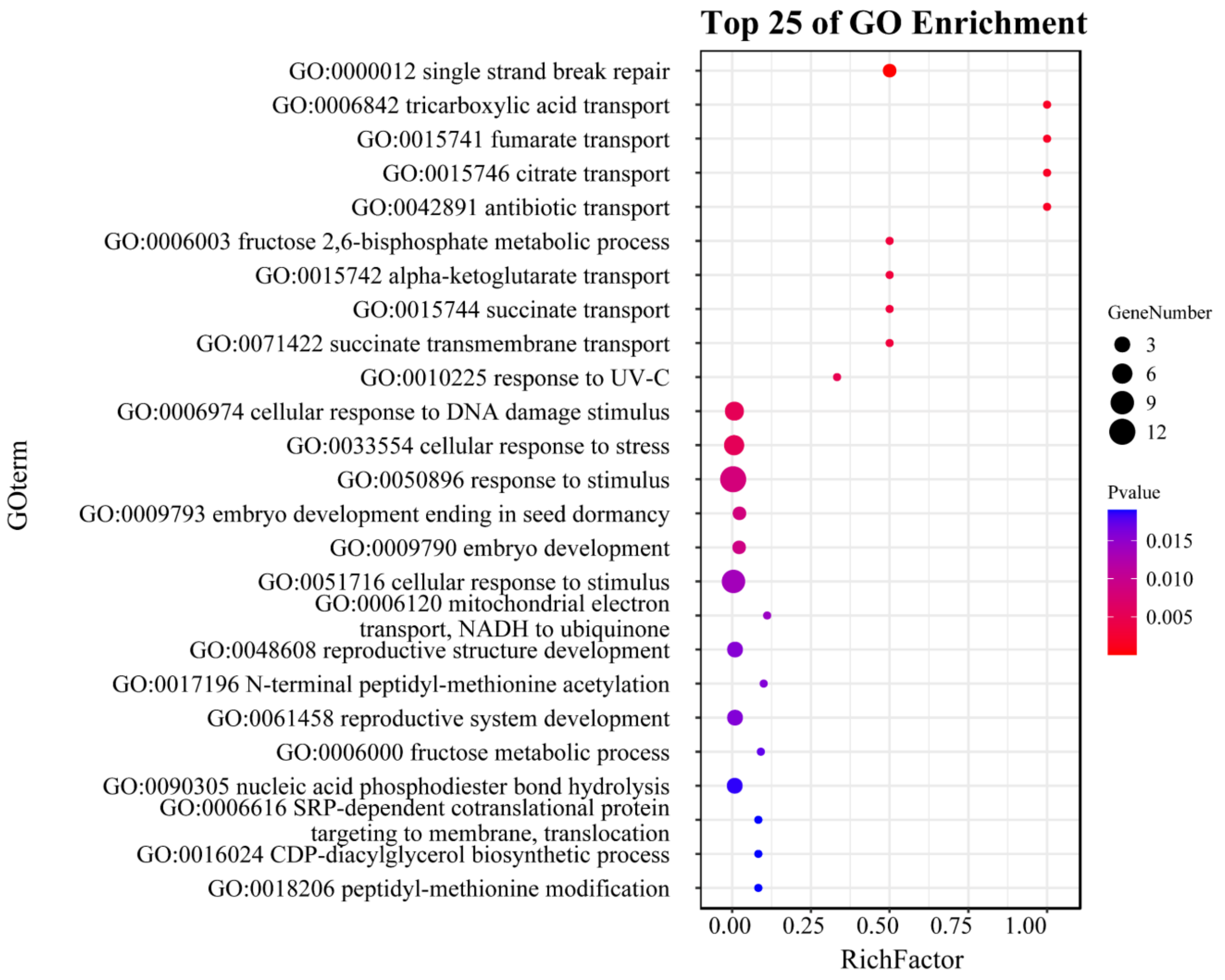

Figure S5: GO term enrichment analysis of 86 candidate genes. The top 25 (a ranking by P-value) significantly enriched GO terms in biological process group.
