# Supplementary figures and images for: A Combination of a Genome-Wide Association Study and a Transcriptome Analysis Reveals circRNAs as New Regulators Involved in the Response to Salt Stress in Maize
Source: Int J Mol Sci. 2022 Aug 28;23(17):9755. doi: 10.3390/ijms23179755 (PMC9456493; doi:10.3390/ijms23179755)

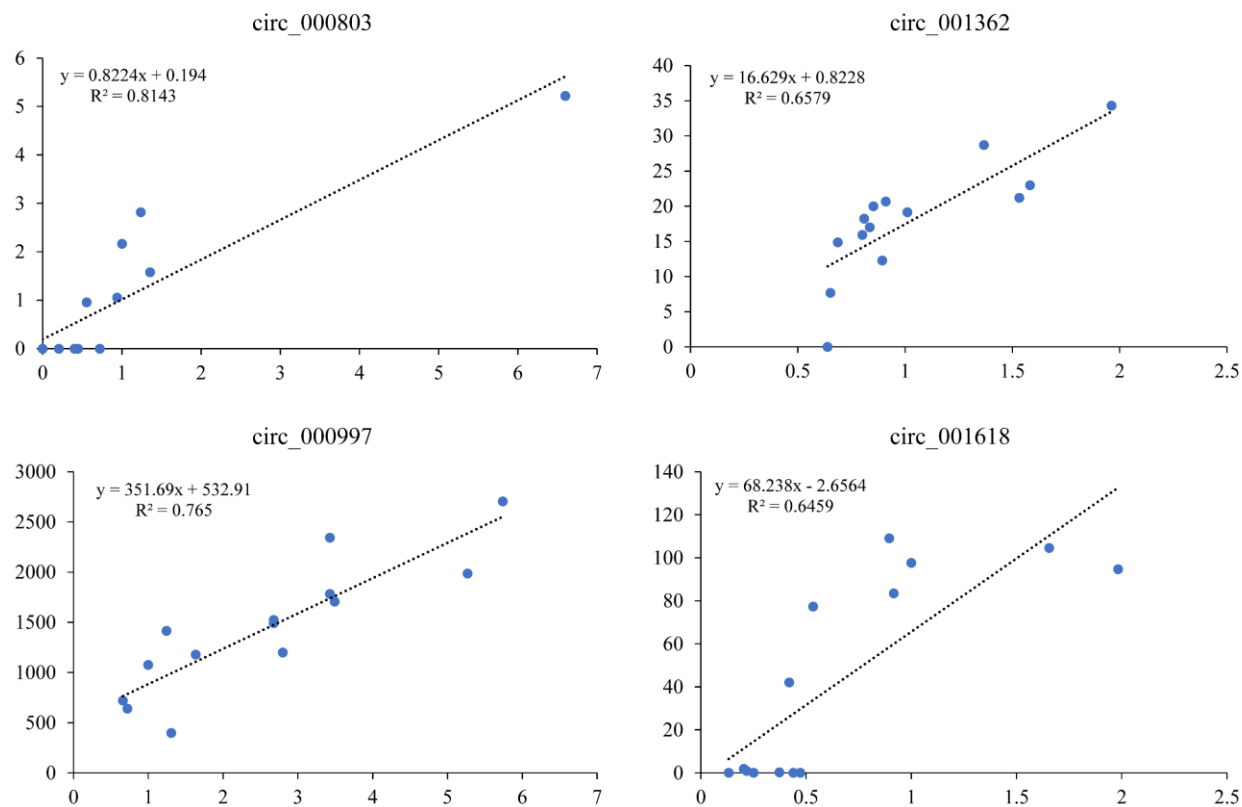

**Figure S1: qRT-PCR for circRNA validation.**

Supplement: Supplementary file 1 [file ijms-23-09755-s001.zip › Figure S1.pdf]

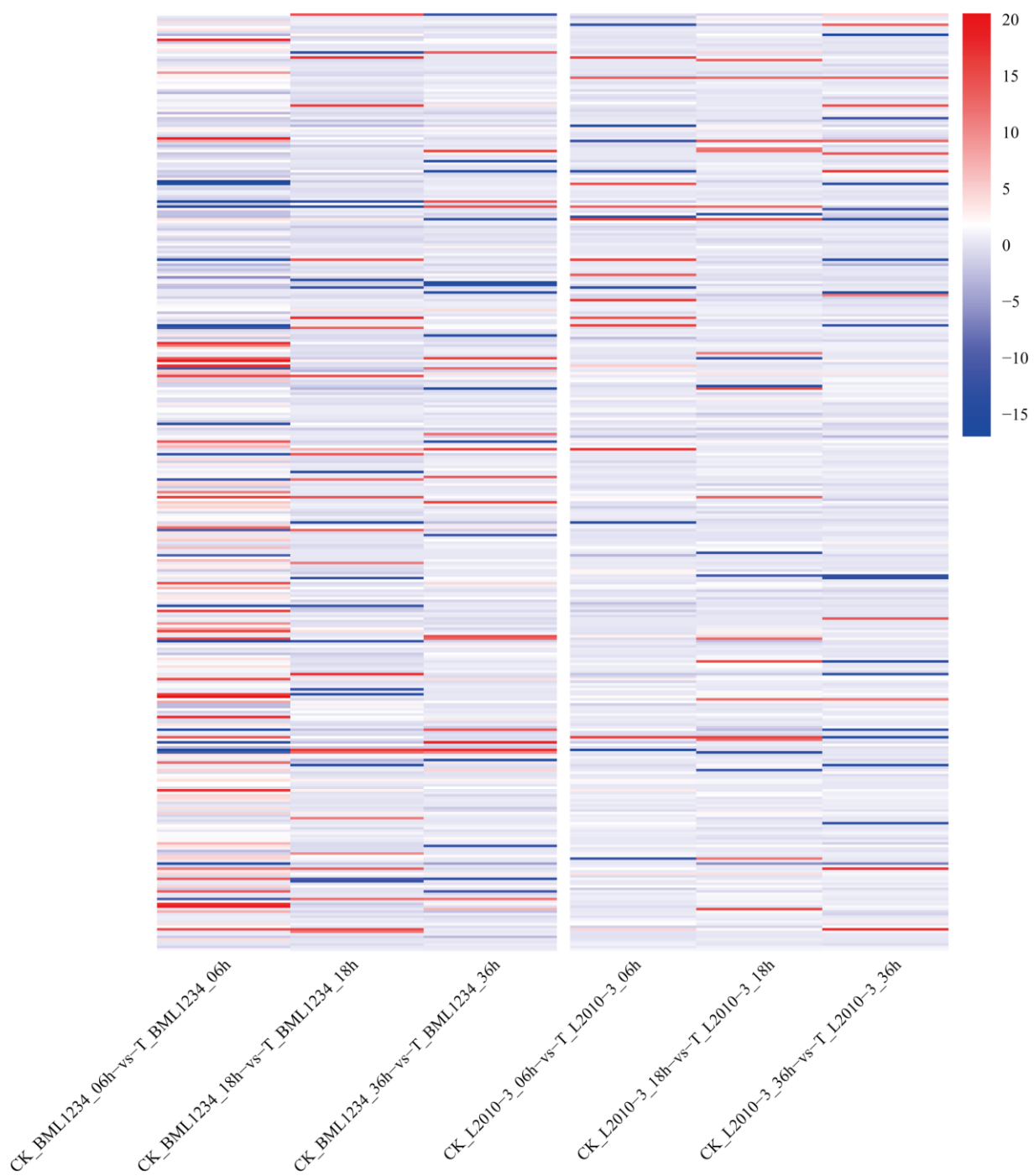

**Figure S2: Heatmap for 371 DECs.**

Supplement: Supplementary file 1 [file ijms-23-09755-s001.zip › Figure S2.pdf]

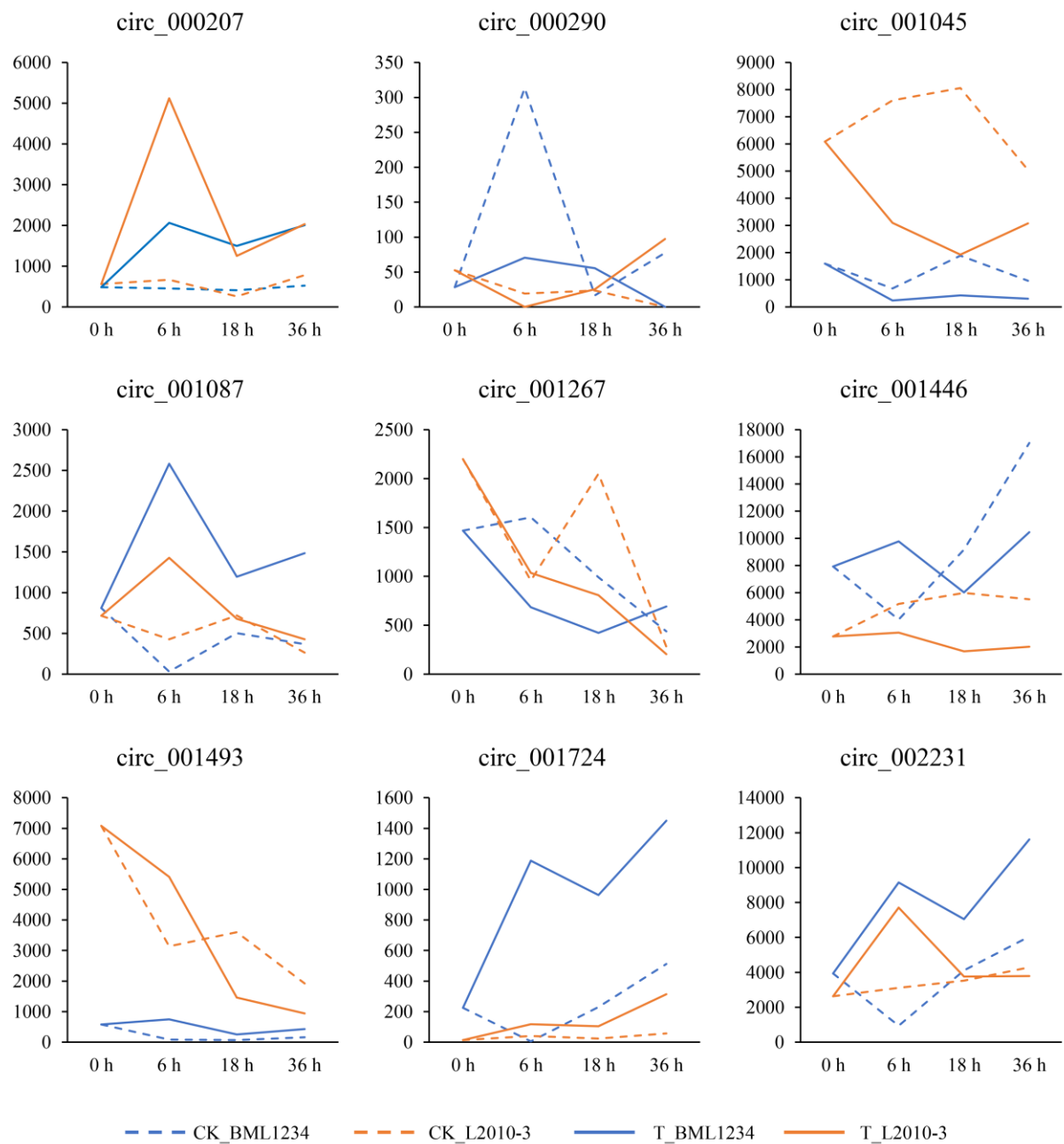

**Figure S3: Expression patterns of nine common DECs.**

Supplement: Supplementary file 1 [file ijms-23-09755-s001.zip › Figure S3.pdf]

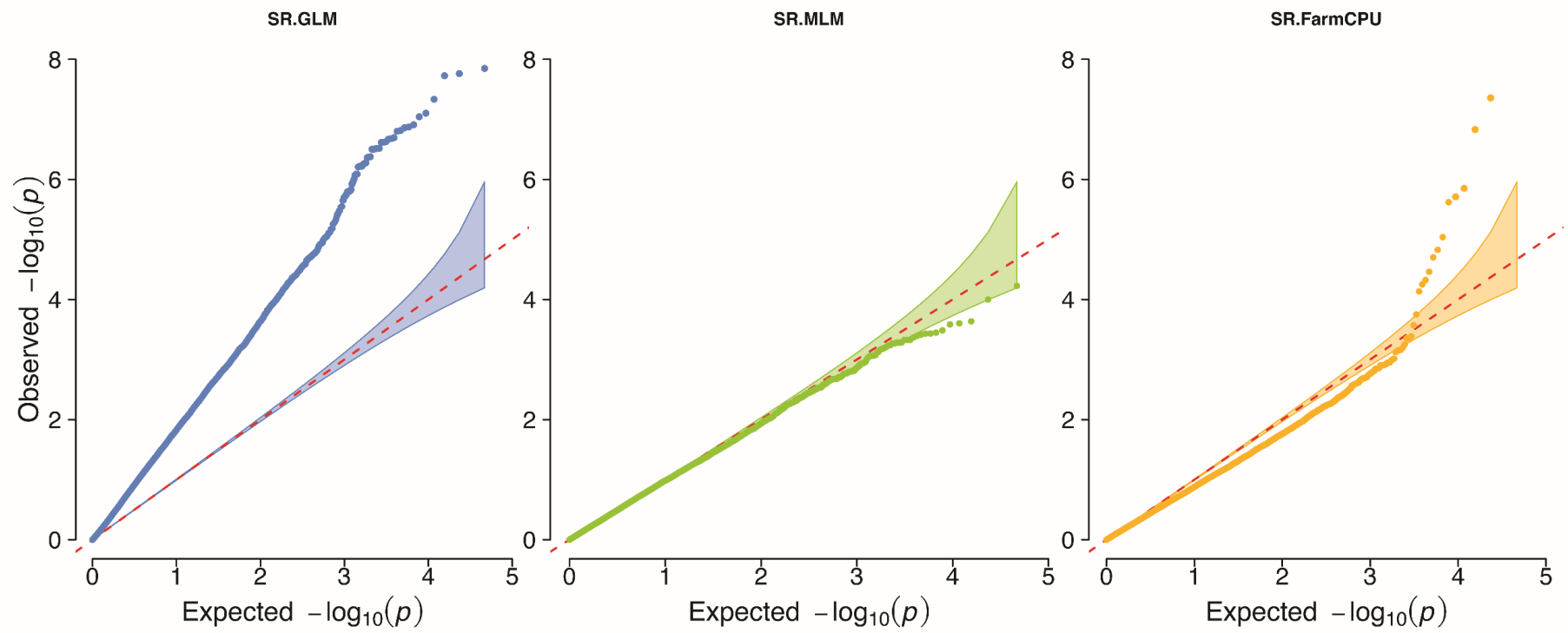

**Figure S4: QQ plot from different models.**

Supplement: Supplementary file 1 [file ijms-23-09755-s001.zip › Figure S4.pdf]

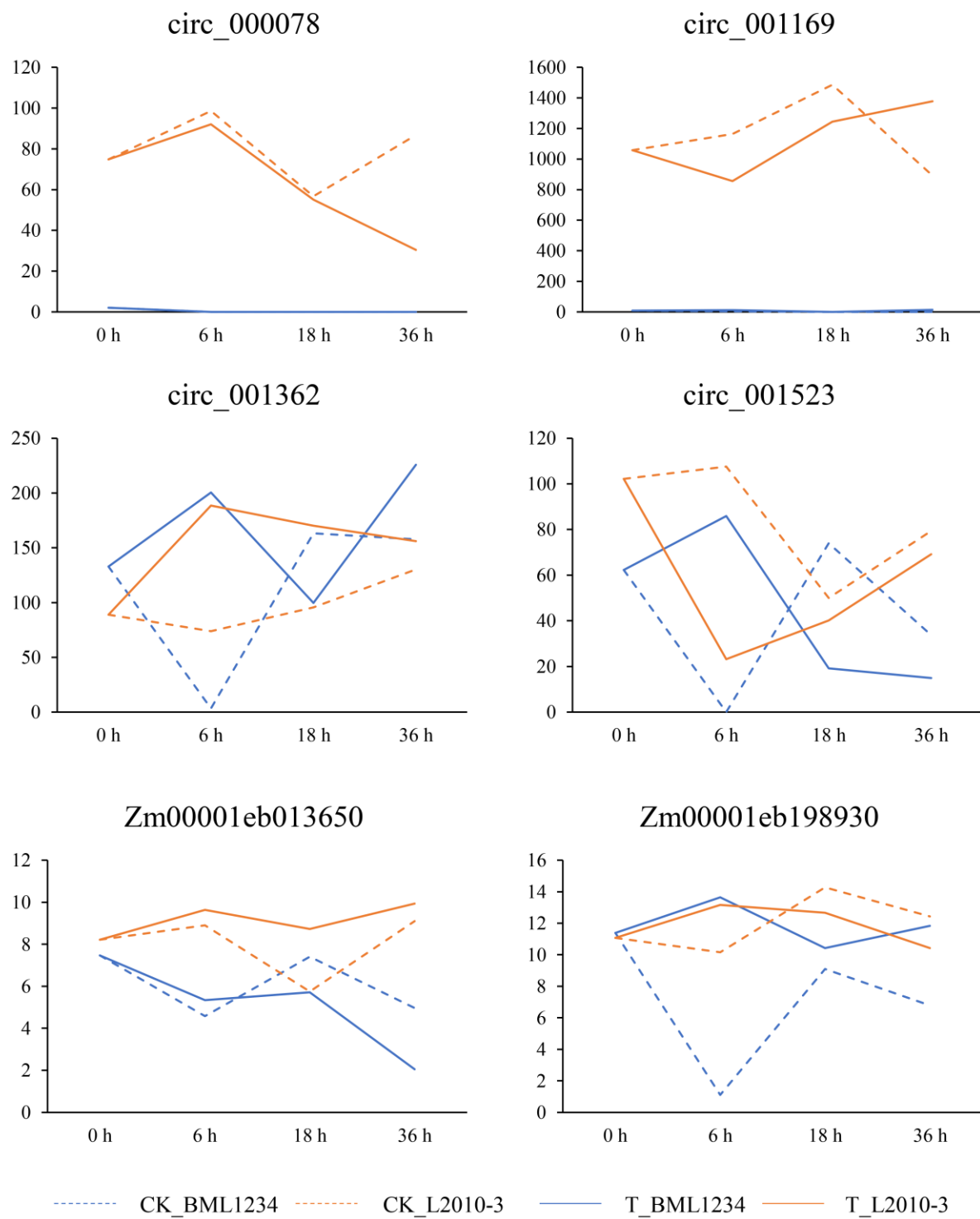

**Figure S6: Expression patterns of four circRNAs and two hub genes.**

Supplement: Supplementary file 1 [file ijms-23-09755-s001.zip › Figure S6.pdf]
